# High expression of ACE2 receptor of 2019-nCoV on the epithelial cells of oral mucosa

**DOI:** 10.1038/s41368-020-0074-x

**Published:** 2020-02-24

**Authors:** Hao Xu, Liang Zhong, Jiaxin Deng, Jiakuan Peng, Hongxia Dan, Xin Zeng, Taiwen Li, Qianming Chen

**Affiliations:** 1 State Key Laboratory of Oral Diseases, National Clinical Research Center for Oral Diseases, Chinese Academy of Medical Sciences Research Unit of Oral Carcinogenesis and Management, West China Hospital of Stomatology, Sichuan University, Chengdu, Sichuan China; 2 Key Laboratory of Oral Biomedical Research of Zhejiang Province, Affiliated Stomatology Hospital, Zhejiang University School of Stomatology, Hangzhou, Zhejiang China

**Keywords:** Biomarkers, Diseases

## Abstract

It has been reported that ACE2 is the main host cell receptor of 2019-nCoV and plays a crucial role in the entry of virus into the cell to cause the final infection. To investigate the potential route of 2019-nCov infection on the mucosa of oral cavity, bulk RNA-seq profiles from two public databases including The Cancer Genome Atlas (TCGA) and Functional Annotation of The Mammalian Genome Cap Analysis of Gene Expression (FANTOM5 CAGE) dataset were collected. RNA-seq profiling data of 13 organ types with para-carcinoma normal tissues from TCGA and 14 organ types with normal tissues from FANTOM5 CAGE were analyzed in order to explore and validate the expression of ACE2 on the mucosa of oral cavity. Further, single-cell transcriptomes from an independent data generated in-house were used to identify and confirm the ACE2-expressing cell composition and proportion in oral cavity. The results demonstrated that the ACE2 expressed on the mucosa of oral cavity. Interestingly, this receptor was highly enriched in epithelial cells of tongue. Preliminarily, those findings have explained the basic mechanism that the oral cavity is a potentially high risk for 2019-nCoV infectious susceptibility and provided a piece of evidence for the future prevention strategy in dental clinical practice as well as daily life.

## Introduction

Since December 2019, an increasing number of patients with pneumonia occurred in Wuhan, Hubei province, China, which attracted much attention not only within China but across the world^1,2^. The novel pneumonia was named as Corona Virus Disease 19 (COVID-19) by World Health Organization (WHO) (https://www.who.int/docs/default-source/coronaviruse/situation-reports/20200211-sitrep-22-ncov.pdf?sfvrsn=fb6d49b1_2), the common symptoms of COVID-19 at illness onset were fever, fatigue, dry cough, myalgia, and dyspnea^3^. In addition, some patients might suffer from headache, dizziness, abdominal pain, diarrhea, nausea, and vomiting^3^. Onset of disease may lead to progressive respiratory failure due to alveolar damage and even death^4^.

Scientists then isolated a novel coronavirus from human airway epithelial cells, which was named 2019-nCoV^5^. Lu et al.^6^ found that 2019-nCoV was closer to bat-SL-CoVZC45 and bat-SL-CoVZXC21 at the whole-genome level, and the external subdomain of the 2019-nCoV receptor-binding domain (RBD) was more similar to that of severe acute respiratory syndrome (SARS) coronavirus (SARS-CoV). Study of Zhou et al.^4^ indicated that the angiotensin-converting enzyme II (ACE2) is likely the cell receptor of 2019-nCoV, which were also the receptor for SARS-CoV and HCoV-NL63^7,8^. Zhou et al.^4^ also proved that 2019-nCoV does not use other coronavirus receptors, aminopeptidase N, and dipeptidyl peptidase 4. The study of Xu et al.^9^ found that the RBD domain of the 2019-nCoV S-protein supports strong interaction with human ACE2 molecules. These findings suggest that the ACE2 plays an important role in cellular entry, thus ACE2-expressing cells may act as target cells and are susceptible to 2019-nCoV infection^10^.

The expression and distribution of the ACE2 in human body may indicate the potential infection routes of 2019-nCoV. Through the developed single-cell RNA sequencing (scRNA-Seq) technique and single-cell transcriptomes based on the public database, researchers analyzed the ACE2 RNA expression profile at single-cell resolution. High ACE2 expression was identified in type II alveolar cells (AT2) of lung^10–12^, esophagus upper and stratified epithelial cells, absorptive enterocytes from ileum and colon^12^, cholangiocytes^13^, myocardial cells, kidney proximal tubule cells, and bladder urothelial cells^10^. These findings indicated that those organs with high ACE2-expressing cells should be considered as potential high risk for 2019-nCoV infection^10^.

In order to investigated the potential routes of 2019-nCov infection on the mucosa of oral cavity, we explored whether the ACE2 is expressed and the ACE2-expressing cell composition and proportion in oral cavity based on the public bulk RNA-seq profiles from two public databases and single-cell transcriptomes from an independent data generated in-house. The result showed that the ACE2 could be expressed in the oral cavity, and was highly enriched in epithelial cells. Moreover, among different oral sites, ACE2 expression was higher in tongue than buccal and gingival tissues. These findings indicate that the mucosa of oral cavity may be a potentially high risk route of 2019-nCov infection.

## Results

### Public bulk RNA-seq dataset analysis

**Fig. 1:**
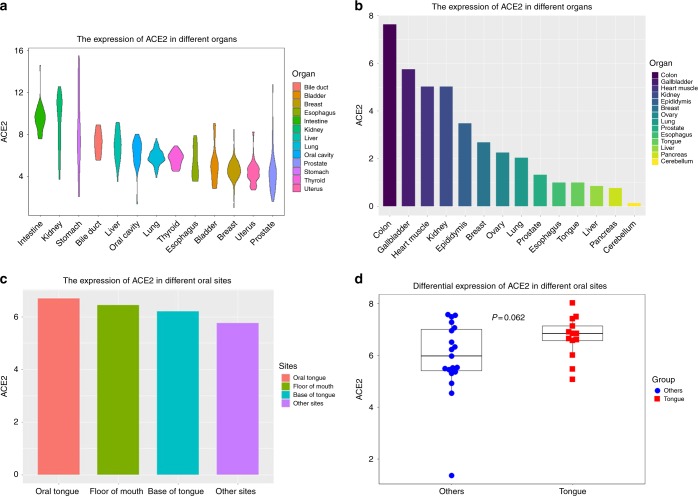
Bulk RNA-seq analysis of public datasets. **a** Violin plot of ACE2 expression in para-carcinoma normal tissues from TCGA, colored by organs. **b** Bar plot of ACE2 expression in normal tissues from FANTOM5 CAGE dataset, colored by organs. **c** Bar plot of ACE2 expression of para-carcinoma normal tissues from TCGA in different oral sites, colored by oral sites; **d** Boxplot of ACE2 in two kinds of oral sites from TCGA, colored by oral sites

NA-seq profile data of 13 organs including 695 para-carcinoma normal tissues as control from public TCGA were obtained for our analysis, and Fig. 1a showed that ACE2 could be expressed in various organs, the mean expression of different organs could be found in Table 1. According to the mean expression of ACE2, the mucosa of oral cavity could express ACE2, and the results were validated by the data of normal tissues from the FANTOM5 CAGE dataset (Fig. 1b).

**Table 1:** Sample size and ACE2 expression of para-carcinoma normal tissues in different organs

	Sample size	ACE2 mean expression	Standard deviation of expression
Intestine	51	9.50	1.183
Kidney	129	9.20	2.410
Stomach	35	8.25	3.715
Bile duct	9	7.23	1.163
Liver	50	6.86	1.351
Oral cavity	32	6.23	1.271
Lung	110	5.83	0.710
Thyroid	59	5.65	0.646
Esophagus	11	5.31	1.552
Bladder	19	5.10	1.809
Breast	113	4.61	0.961
Uterus	25	4.37	1.125
Prostate	52	4.35	1.905

To investigate the ACE2 expression on mucosa of oral cavity, we looked into the ACE2 expression in different oral sites. According to the site information provided by the TCGA, among the 32 adjacent normal tissues, 13 tissues located in the oral tongue, 2 tissues located in the base of tongue, 3 tissues located in the floor of mouse, and 14 tissues did not definite the site and were just put into the category of oral cavity. The mean expression distribution of different sites was shown in Fig. 1c. When we combined the base of tongue, floor of mouth and oral cavity as other sites, and compared them with oral tongue, we found the obvious tendency that the mean expression of ACE2 was higher in oral tongue (13 tissues) than others (19 tissues) (Fig. 1d), while may due to the limitation of the sample size, the *p* value was not significant (*P* = 0.062).

### Single cell RNA-seq analysis of oral tissues

**Fig. 2:**
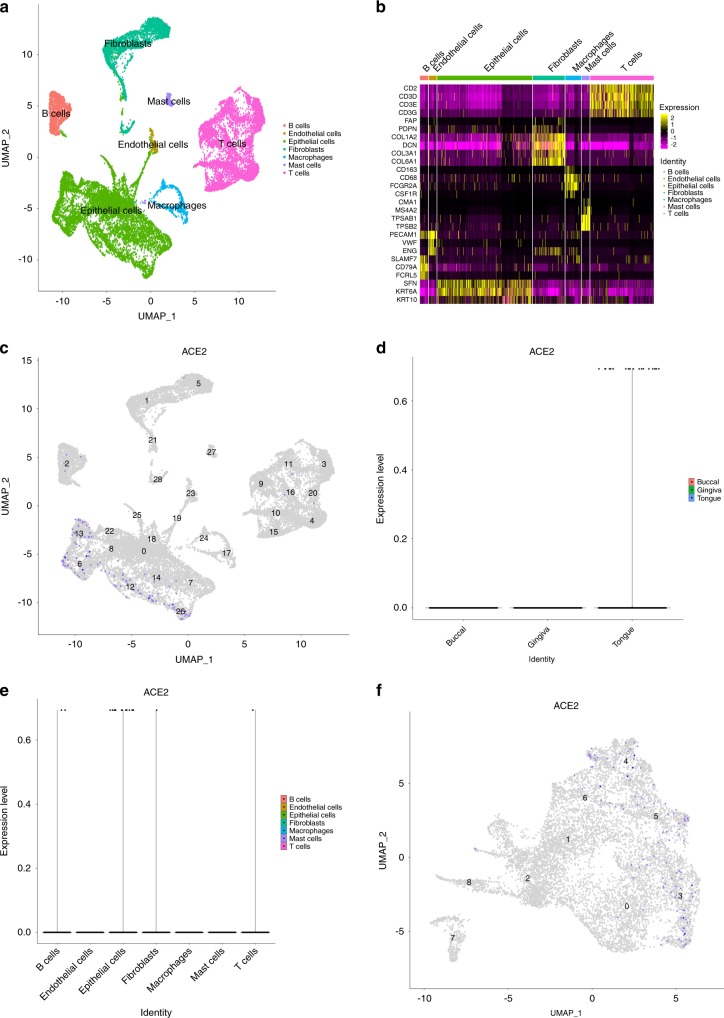
Single cell RNA-seq analysis of oral tissues from independent dataset. **a** Seven-cell types were identified by the cell markers; cells were clustered by UMAP method. **b** Heatmap of cell markers for identifying cell types. **c** Scatter plots of all the cells with ACE2 expression. **d** Violin plot of ACE2 expression across different oral sites. **e** Violin plot of ACE2 expression across different cell types. **f** Scatter plots of tongue epithelial cells with ACE2 expression

Single cell RNA-seq was utilized for four oral tissues, and the data was analyzed to confirm the above results and assess the cell type-specific expression of ACE2. After the data preprocessing (shown in section “Materials and methods”), 22 969 cells were acquired and 7 cell types were identified (Fig. 2a), including epithelial cells (marker genes including SFN, KRT6A, and KRT10), fibroblasts (marker genes including FAP, PDPN, COL1A2, DCN, COL3A1, COL6A1), T cells (marker genes including CD2, CD3D, CD3E, and CD3G), macrophages (marker genes including CD163, CSF1R, CD68, and FCGR2A), mast cells (marker genes including CMA1, MS4A2, TPSAB1, TPSB2), B cells (marker genes including SLAMF7, FCRL5, and CD79A) and endothelial cells (marker genes including PECAM1, VWF, and ENG). The heatmap of main cell markers across the cell types can be found in Fig. 2b.

According to Fig. 2c, d, we confirmed the ACE2 was expressed in oral tissues (0.52% ACE2-positive cells), and higher in oral tongue than buccal and gingival tissues (95.86% ACE2-positive cells located in oral tongue). Figure 2e shows that the ACE2-positive cells could be found in oral tissues including epithelial cells (1.19% ACE2-positive cells), T cells (<0.5%), B cells (<0.5%), and fibroblast (<0.5%), and the ACE2 was highly enriched in epithelial cells, of which 93.38% ACE2-positive cells belong to epithelial cells (Fig. 2f). The above results indicated that the ACE2 could be expressed on the epithelial cells of the oral mucosa and highly enriched in tongue epithelial cells.

## Discussion

In the last two decades, coronavirus has caused two large-scale pandemics, SARS in 2002 and the Middle East respiratory syndrome (MERS) in 2012^14^. In December 2019, a novel coronavirus (2019-nCov) induced an outbreak of pneumonia in Wuhan, China, restated the risk of coronaviruses posed to public health^15^. The infection routes and pathogenesis of 2019-nCov are not fully understood by far, and the study of 2019-nCoV host cell receptor ACE2 could be valuable for the prevention and treatment of the COVID-19.

In this study, the analysis of public bulk-seq RNA datasets showed that the mucosa of oral cavity could express the ACE2 and was higher in tongue than other oral sites. The results of this study were consistent with the study of Zou et al.^10^ in general, many organs with higher expression of ACE2 than lung, such as intestine, heart, and kidney. According to the study of Zhao et al.^11^, the ACE2 expression in lung is concentrated in a small population of type II alveolar cells (AT2), that may cause the relatively low ACE2 expression of lung in bulk-seq RNA datasets analysis. Even though, the result of Zou et al. indicated that the respiratory tract should also be considered as a vulnerable target to 2019-nCoV infection^10^.

The results of our single cell RNA-seq profiles validated the ACE2 expression in oral cavity, and the level of ACE2 expression in oral tissues was higher in tongue than buccal or gingival tissues. Furthermore, we have also demonstrated that the ACE2-positive cells were enriched in epithelial cells, which was also reported by previous study^10–12,16^. These findings indicated that oral cavity could be regarded as potentially high risk for 2019-nCov infectious susceptibility.

Interestingly, we found that the ACE2 also expressed in lymphocytes within oral mucosa, and similar results were found in various organs of the digestive system and in lungs^11,12^. Whether those facts have reminded the 2019-nCoV attacks the lymphocytes and leads to the severe illness of patients needs more in vitro and in vivo evidence and validations, though the proportion of ACE2-positive lymphocytes is quite small.

Previous studies have investigated the ACE2 mRNA and protein expression in various tissues by bulk samples^17,18^, however, the distribution of ACE2 through bulk data could not indicate the cell type-specific expression of ACE2. Recently developed single-cell RNA-sequencing technology enabled the generation of vast amounts of the transcriptomic data at cellular resolution^19^. The ACE2 expression profile in various organs, tissues, and cell types, provides the bioinformatics evidence for the potential infection routes of 2019-nCov, which might also be associated with presented symptoms.

Although studies have reported multiple symptoms of hospitalized patients with 2019-nCoV infection^3,20^, some cases at home might be asymptomatic. It is worth noting that, a previous study showed that 99% of the patients had no clinical manifestation of oral human papillomavirus (HPV), but HPV DNA was detected in 81% of oral mucosa samples, and anti-HPV IgA was detected in the saliva of 44% of the patients^21^. Likewise, although 2019-ncov infection hardly presented oral symptoms, the ACE2 expression in the oral cavity indicated that the oral infection route of 2019-nCoV cannot be excluded. Moreover, a latest pilot experiment showed that 4 out of 62 stool specimens tested positive to 2019-nCoV, and another four patients in a separate cohort who tested positive to rectal swabs had the 2019-nCoV being detected in the gastrointestinal tract, saliva, or urine^20^. Thus, our results support that in addition to the respiratory droplets and direct contact, fecal–oral transmission might also be the route of transmission of 2019-nCoV.

Our results are mainly based on public datasets and single cell RNA-sequencing data of in-house oral tissues with minimal diseased lesion which from our previous project found no significant expression difference among the common epithelial markers in our past study and other previous study^22–24^. It is warrant that further histological methods are used to confirm our results and enhance the persuasion of the conclusion.

The ACE2-expressing cells in oral tissues, especially in epithelial cells of tongue, might provide possible routes of entry for the 2019-nCov, which indicate oral cavity might be a potential risk route of 2019-nCov infection. Those preliminary findings have explained the basic mechanism that the oral cavity is a potentially high risk for 2019-nCoV infectious susceptibility and provide a piece of evidence for the future prevention strategy in clinical practice as well as daily life.

## Materials and methods

### Public datasets acquisition and analysis

Bulk RNA-seq data of para-carcinoma normal tissues which were taken as control tissues in the studies were downloaded from The Cancer Genome Atlas (TCGA; https://www.cancer.gov/tcga), and 695 para-carcinoma normal tissues distributed in different organs were obtained for this study, which included intestine (51 tissues), kidney (129 tissues), stomach (35 tissues), bile duct (9 tissues), liver (50 tissues), oral cavity (32 tissues), lung (110 tissues), thyroid (59 tissues), esophagus (11 tissues), bladder (19 tissues), breast (113 tissues), uterus (25 tissues), and prostate (52 tissues). The RNA-seq data were batch effects normalized and log2-transformed for the subsequent analysis. Violin plot was used to show the distribution of ACE2 expression among different organs, *t* test was performed to compare the ACE2 expression between two different groups shown in boxplot.

Besides, Bulk RNA-seq data of normal tissues were downloaded from Functional Annotation of The Mammalian Genome Cap Analysis of Gene Expression (FANTOM5 CAGE) dataset^25^, as only two samples of this dataset which owns 60 samples in total located in the tongue, we just downloaded 14 organ types to validate the ACE2 expression in oral cavity with bar plot, including colon, ovary, breast, cerebellum, epididymis, esophagus, gallbladder, heart muscle, kidney, liver, lung, pancreas, prostate, and tongue.

All analyses were performed in R (R version 3.6.0) and significant level was set as 0.05.

### Oral tissues of in-house cohort

Due to our previous project about oral potential malignant disorders, four tissues of oral mucosa were obtained from patients after informed consent and ethical approval from West China Hospital of Stomatology, Sichuan University. These oral tissues had been sent for single cell RNA sequence. The four were taken from three patients with an average age of 50, which were all diagnosed as hyperkeratosis without dysplasia by pathologists, just showing an increase in cell number in the spinous layer and/or in the basal/parabasal cell layers without cellular atypia, and its genetic profiles would be much more closed to normal tissue than malignant tissue^22–24^. Two tissues were from independent lesions of one patient’s dorsum linguae. The other two tissues came from buccal and gingival sites.

### Tissue dissociation from fresh biopsies

Fresh biopsy tissues were washed twice by D-PBS (Hyclone) and collected into prechilled MACS Tissue Storage Solution (Miltenyi). Single-cell suspensions were generated from biopsy tissues using Whole Skin Dissociation Kit human (Miltenyi) manufacturer guidelines and filtered by 70 μm MACS Smartstrainers (Miltenyi). Carryover red blood cells were lysed by Red Blood Cell Lysis Buffer (Abcam) and dead cells were removed by Easysep Dead Cell Removal (Annexin V) Kit (STEMCELL). Finally, cell pellets were re-suspended in D-PBS.

### Single cell RNA-sequencing library preparation

To generate single-cell Gel Beads-in-Emulsion (GEMs), the 10× chromium platform was used to capture and barcode cells. Cells were partitioned into GEMs along with gel beads coated with oligonucleotides and cDNAs with both barcodes were amplified, and a library was constructed using the 10× Genomics Chromium Single Cell Kit (v3 chemistry) for each sample. The resulting libraries were sequenced on an Illumina NovaSeq 6000 System.

### Single cell RNA-seq data preprocessing and analysis

The FASTQ files were analyzed with the Cell Ranger Software Suite (version 3.1; 10× Genomics). The Seurat (version 3.0) was applied to read the gene-barcode matrix of four tissues. To control quality, we removed cells with <200 genes and 500 UMI counts, and as well as the cells with mitochondrial content higher than 5%. Besides, the genes detected in <3 cells were filtered out. The “sctransform” wrapper in Seurat was applied to normalize the data and remove confounding sources of variation, the “IntegrateData” was used for integrated the Seurat objects from four tissues. The uniform manifold approximation and projection (UMAP) was used for dimensionality reduction and clustering the cells, cell types were assigned based on their canonical markers. UMAP plots, heatmap, and violin plots were generated with Seurat in R.
